# Dynamic changes of the fecal bacterial community in dairy cows during early lactation

**DOI:** 10.1186/s13568-020-01106-3

**Published:** 2020-09-17

**Authors:** Shuai Huang, Shoukun Ji, Feiran Wang, Jie Huang, Gibson Maswayi Alugongo, Shengli Li

**Affiliations:** 1 The State Key Laboratory of Animal Nutrition, Beijing Engineering Technology Research Center of Raw Milk Quality and Safety Control, College of Animal Science and Technology, China Agricultural University, Beijing, 100193 China; 2 College of Animal Science and Technology, Hebei Agricultural University, Baoding, 071001 China; 3 College of Animal Science and Technology, Yangzhou University, Yangzhou, 225009 China

**Keywords:** Fecal microbiota, Fresh dairy cows, Dynamic, 16S rRNA sequencing

## Abstract

The dynamics of the community structure and composition of the dairy cow fecal bacterial communities during early lactation is unclear, therefore this study was conducted to characterize the fecal bacterial communities in dairy cows during early lactation using 16S rRNA gene sequencing. Feces were sampled from 20 healthy fresh Holstein dairy cows on day 1 (Fresh1d group) and day 14 (Fresh14d group) after calving. After calving, cows were fed the same fresh diet. The dominant phyla *Firmicutes* and *Proteobacteria* were decreased (*P* ≤ 0.01) with lactating progress and phyla *Bacteroidetes* were increased (*P* = 0.008) with lactating progress and dietary transition. At family level, the predominant families were *Ruminococcaceae* (35.23%), *Lachnospiraceae* (11.46%), *Rikenellaceae* (10.44%) and *Prevotellaceae* (6.89%). A total of 14 genera were different between fecal samples from Fresh1d and Fresh14d, included the predominant genera, such as *Ruminococcaceae_UCG*-*005* (*P *= 0.008), *Rikenellaceae_RC9_gut_group* (*P *= 0.043) and *Christensenellaceae_R*-*7_group* (*P *= 0.008). All fecal bacterial communities shared members of the genera *Ruminococcaceae_UCG*-*005*, *Bacteroides* and *Rikenellaceae_RC9_gut_group*. These findings help to improve our understanding of the composition and structure of the fecal microbial community in fresh cows and may provide insight into bacterial adaptation time and dietary in lactating cows.

## Introduction

The bovine gastrointestinal tract microbiota harbors lots of microbial species that play important roles in the health and productivity of ruminant (Bergmann 2017; Clemmons et al. 2019; Myer et al. 2015). These microbes are necessary for fermentation of ingested plant matter into compounds such as volatile fatty acids that act as energy sources for the host (Flint et al. 2008). The development of high-throughput sequencing technology has enabled advancements in the understanding of the gastrointestinal microbiota of ruminant animals in recent years. Studies on the microbiota present in rumen during lactation have found that the ruminal microbial structure and composition varied with lactation period (Bainbridge et al. 2016; Lima et al. 2015; Xue et al. 2018), and revealed an association between the rumen microbiota and milk efficiency (Weimer et al. 2017), feed efficiency (Shabat et al. 2016) and milk production (Indugu et al. 2017; Tong et al. 2018) in dairy cows.

In addition to studies on the rumen, reports on the microbial composition of the feces in dairy calves are abundant. Previous studies have found an association between fecal microbiota and age (Song et al. 2018), diet (Dill-McFarland et al. 2019; Wang et al. 2019), antibiotic therapy (Behr et al. 2018; Oultram et al. 2015; Yousif et al. 2018), and health (Gomez et al. 2017) in dairy calves. For lactating cows, the core fecal microbiota was identified from ten farms across Northern and Central California, USA (Hagey et al. 2019). Another study compared the fecal microbiota of health and left-sided displacement of the abomasum and found a shift in fecal microbiota composition with left-sided displacement in early lactating dairy cows (Song et al. 2016). Therefore, to date, the knowledge on the fecal microbiota structure and composition during early lactation stage in dairy cows, remain sparse.

It is well known that diet can make a significantly influence in the structure of the fecal microbiome (Kim et al. 2014; Zhang et al. 2018). However, lactation was found to be another important factor to shape rumen microbiome in dairy cows (Bainbridge et al. 2016). For fecal microbiota, limited knowledge about the diet and lactating effect on fecal microbiota. Therefore, elucidating the dynamic changes of fecal microbiota in dairy cows is expected to enable improvements to feed and management strategies for dairy cows, especially in the fresh period, which is the most sensitive window for dairy cows. Here, we aimed to characterize the community structure and composition of the fecal microbiota in dairy cows during early lactation stage. Our findings may contribute to the state of knowledge on the hindgut bacterial community structure in lactating dairy cows and identify genera of interest for further studies into the functional roles of the fecal microbiota in the health of the host.

## Materials and methods

### Cows and management

Twenty fresh (2.48 ± 0.59 parity) Holstein dairy cows were housed in a free-stall barn at a commercial dairy farm (Beijing, China). The dietary and nutritional composition of the feed given to fresh cows is presented in Additional file 1: Table S1. Cows were allowed ad libitum access to feed and fresh water.

### Collection of fecal samples

Forty fecal samples were collected from 20 healthy fresh cows on d1 and d14 after calving, without a history of antibiotic or drug treatment for 3 months prior to collecting samples. Feces were collected by hand from the rectum of cows using sterilized gloves before morning feeding. All samples were immediately transported on liquid nitrogen and later stored at − 80 °C before DNA extraction.

### Fecal bacteria DNA extraction, amplification and sequencing

Total DNA was extracted from all fecal samples using an Omega Stool DNA kit (Omega Bio-Tek, Norcross, GA, USA) according to the manufacturer’s instructions. Amplicon library preparation was performed by PCR amplification of the V3 to V4 region of the 16S rRNA gene, using forward (338F, 5′-ACTCCTACGGGAGGCAGCAG-3′) and reverse primers (306R, 5′-GGACTACHVGGGTWTCTAAT-3′). Then, the amplicon library was sequenced using the Illumina Miseq platform (Illumina, San Diego, CA, USA) by Beijing Allwegene Tech. Ltd (Beijing, China). A 25 µL reaction mixture containing 12.5 μL of KAPA 2G Robust Hot Start Ready Mix (Kapa Biosystems, Wilmington, MA, USA), 1 µL of each primer (5 µM), 30 ng of template DNA and 5.5 µL ddH_2_O was used for PCR in triplicate, with the following cycling conditions: 95 °C for 5 min in denaturation, followed by 28 cycles of 95 °C for 45 s in denaturation, 55 °C for 50 s in annealing and 72 °C for 45 s in elongation at with a final extension at 72 °C for 10 min.

Amplicons were detected by 1% agarose gel electrophoresis and purified using the Agencourt AM Pure XP Kit (Beckman Coulter Genomics, Indianapolis, IN, USA). Purified amplicons from samples were quantified using Caliper LabChip GX Touch HT (PerkinElmer, Downers Grove, IL, USA), pooled in equimolar concentrations into the final library, and then 2 × 250 paired-end sequenced on an Illumina MiSeq platform (Caporaso et al. 2012).

### Cleanup of sequencing data

High-quality sequence extraction was first conducted with Quantitative Insights into Microbial Ecology (QIIME) version 1.8.0 (Caporaso et al. 2010). Raw FASTQ files were de-multiplexed and quality-filtered with the following criteria: (I) reads with an average quality score of less than 20 were removed; (II) reads that did not exactly match to primer sequences and barcode tags and reads containing ambiguous characters were removed; (III) only overlapping sequences longer than 10 bp, and reads less than 230 bp after overlapping were assembled; (IV) reads that could not be assembled were removed. The sequences were classified into operational taxonomic units (OTUs) under the threshold of 97% identity using USEARCH (version 10.0.240) after removing singletons, and chimers were identified and removed using UCHIME (Edgar 2013). The most abundant sequence within each OTU from specific libraries (libraries constructed for bacteria) was designated as the “representative sequence”; this sequence was then aligned against the SILVA 128 16S rRNA gene database (Pruesse et al. 2007), with a confidence threshold of 70%, using the Ribosomal Database Project Classifier (Wang et al. 2007).

### Statistical analysis of microbiota

Alpha diversity indices (Chao1 value, number of OTUs, Shannon and Simpson indices) were assessed with QIIME 1.8. Differences in community richness and diversity were analyzed with the Kruskal–Wallis test, with *P* values corrected by false discovery rate (FDR) from multiple comparisons in package “ggpurb” of R 3.6.2 (R, Armonk, NY, USA). The none-metric multidimensional scaling (NMDS) plot of the microbial profiles was performed based on Bray–Curtis distance (calculated by QIIME) using the “vegan” package in R (Oksanen et al. 2015). Analysis of similarity (ANOSIM) for multivariate data was calculated using the Bray–Curtis distance metric within the function of “vegdist” and “anosim” in the R package “vegan”.

Comparison of the microbial structure and composition at phylum and genus levels were performed using the Wilcoxon tests with the FDR correlation, as described by Benjamini and Hochberg (1995) for pairwise comparisons. The false discovery rate corrected *P* values ≤ 0.05 were considered to indicate significant differences.

### Nucleotide sequence accession numbers

All the raw DNA sequences were deposited in the National Center for Biotechnology Information Sequence Read Archive database and are publicly accessible under the accession number PRJNA628713.

## Results

### Sequencing, evenness and richness of the fecal microbiota

Bacterial amplicons for all fecal samples were sequenced, and a total of 1,100,195 raw reads were generated; of these, 1,076,491 high-quality reads were obtained from all the 40 samples. After sub-sampling and clustering, 1000 ± 144 OTUs were identified. The Good’s coverage for each sample was deemed sufficient, with values > 99.00% for all bacterial communities, implying that the current sequencing depth was sufficient to be representative of the microbiota studied (Table 1). During early lactation stage, a significant decrease (*P* < 0.05) in OTU number and Chao 1 was observed from d1 to d14, indicating significant changes in the diversity and richness of fecal microbiota as lactation progressed (Table 1).

**Table 1:** Number of operational taxonomic units (OTU), Good’s coverage, Chao1 and Shannon indices for fecal samples obtained at d 1 and d14 in fresh cows after calving

Indices	Stage		SEM	*P* value
	Fresh1d	Fresh14d		
OTU	1060	940	22.38	0.026
Good’s coverage	0.991	0.992	0.0002	0.026
Chao1	1254.05	1125.12	25.30	0.026
Shannon index	7.76	7.61	0.07	0.440

Fresh1d indicates fecal microbiota samples from cows on d1, Fresh14d indicates fecal microbiota samples from cows on d14

**Fig. 1:**
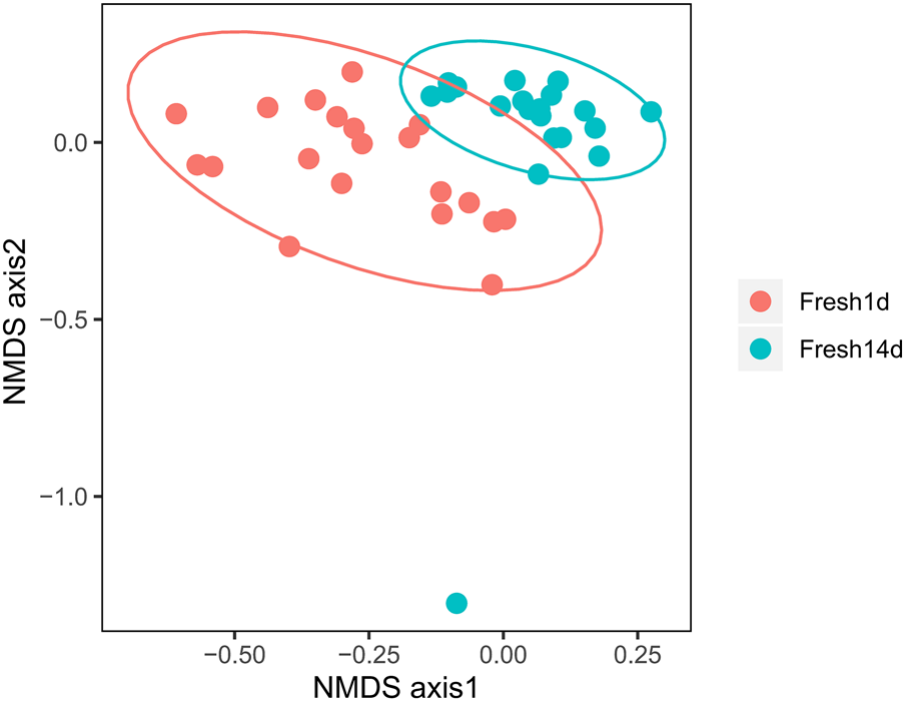
None-metric multidimensional scale analysis based on Bray–Curtis distance. ANOSIM analysis of the samples on d1 and d14, where bacterial communities in dairy cow feces are grouped by lactation period in feces. Analysis was conducted using a Bray–Curtis metric based on operational taxonomic units (R^2^ = 0.447, *P* = 0.001). Fresh1d indicates fecal microbiota samples from cows on d1, Fresh14d indicates fecal microbiota samples from cows on d14

NMDS based on Bray–Curtis distance showed a separation between Fresh1d and Fresh14d groups (Fig. 1). We further performed ANOSIM to demonstrate the effect of the lactation stage. The ANOSIM results revealed a significant difference in fecal bacterial community composition between Fresh1d and Fresh14d (R^2^ = 0.447, *P *= 0.001).

### Taxonomic composition of the fecal microbiota

**Fig. 2:**
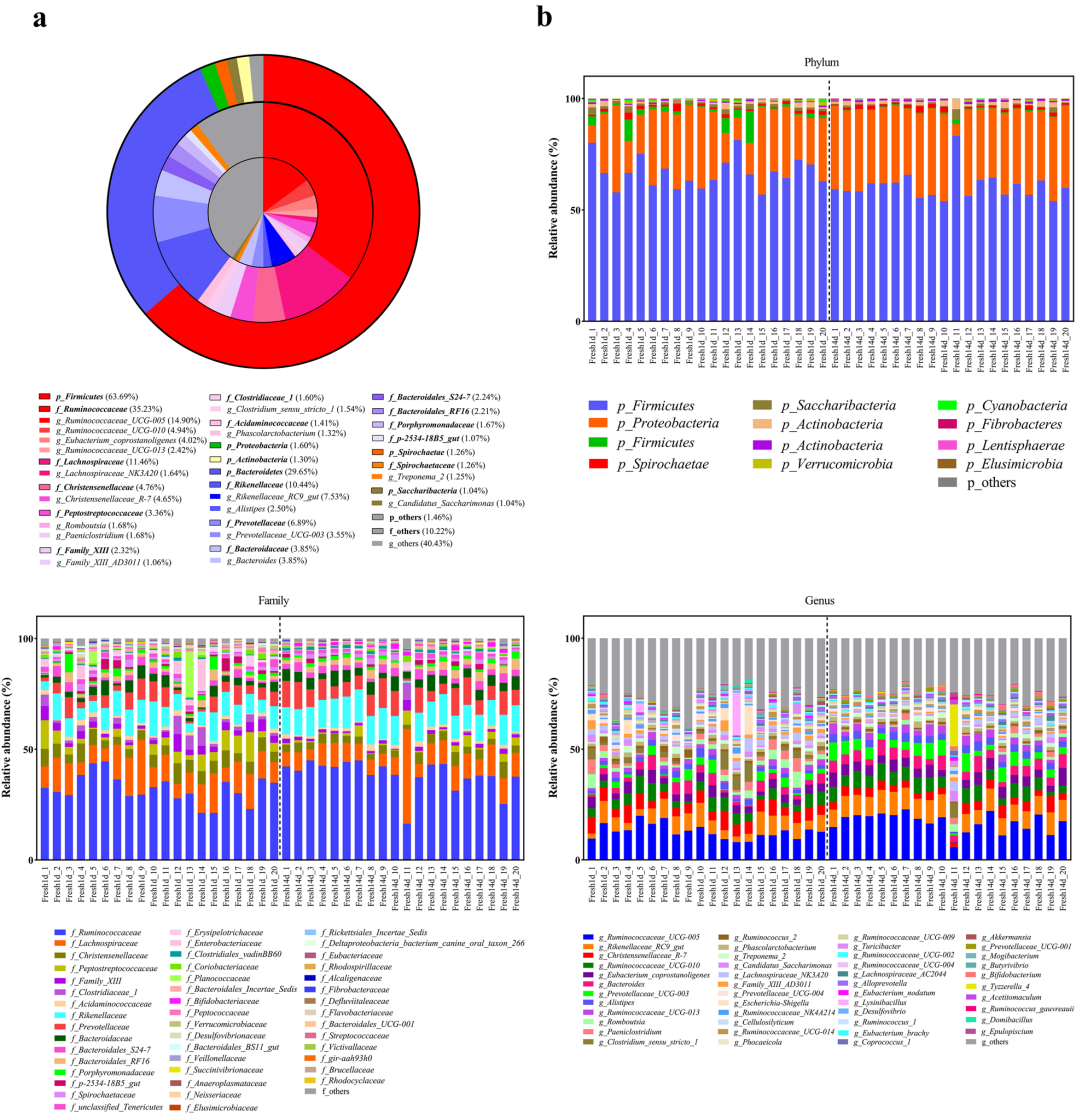
The relative abundance of fecal samples at the phylum, family and genus level. **a** Distribution of predominant phyla, family and genera of all fecal samples (relative abundance > 1% at all samples). **b** The fecal bacterial community composition of all animals at the phyla, family and genus level (relative abundance > 1% at least one sample). Fresh1d indicates fecal microbiota samples from cows on d1, Fresh14d indicates fecal microbiota samples from cows on d14

In total, 17 phyla were identified; among these, *Firmicutes*, *Bacteroidetes*, *Proteobacteria* and *Spirochaetae* were the predominant phyla across all samples, representing 63.69, 29.65, 1.60 and 1.26% of total sequences, respectively (Fig. 2a). Two other phyla, namely *Actinobacteria* and *Saccharibacteria*, accounted for 2.34% of the community and were considered minor contributing phyla (Fig. 2a, b, Table 2).

**Table 2:** Effect of lactation period on diversity at the phylum level in the fecal bacterial community

Phyla	Stage		SEM	*P* value
	Fresh1d	Fresh14d		
*Firmicutes*	66.70	60.68	0.06	0.010
*Bacteroidetes*	25.38	33.93	1.52	0.008
*Proteobacteria*	2.73	0.46	0.01	0.003
*Saccharibacteria*	1.30	0.78	< 0.01	0.025
*Spirochaetae*	1.21	1.32	< 0.01	0.780
*Actinobacteria*	1.08	1.51	0.91	0.067
*Verrucomicrobia*	0.54	0.26	< 0.01	0.067
*Tenericutes*	0.52	0.84	< 0.01	0.003
*Cyanobacteria*	0.32	0.09	0.09	0.013
*Fibrobacteres*	0.09	0.10	0.05	0.380

Fresh1d indicates fecal microbiota samples from cows on d1, Fresh14d indicates fecal microbiota samples from cows on d14

Among all identified families, the predominant family included *Ruminococcaceae* (35.23%), *Lachnospiraceae* (11.46%), *Rikenellaceae* (10.44%) and *Prevotellaceae* (6.89%, Fig. 2a, b). The relative abundance > 2% across samples were *Christensenellaceae* (4.76%), *Bacteroidaceae* (3.85%), *Peptostreptococcaceae* (3.36%), *Family_XIII* (2.32%), *Bacteroidales_S24*-*7_group* (2.24%) and *Bacteroidales_RF16_group* (2.21%), and altogether comprised 18.74% of total samples.

Of the 217 genera identified, *Ruminococcaceae_UCG*-*005* (14.90%), *Rikenellaceae_RC9_gut_group* (7.52%), *Ruminococcaceae_UCG*-*010* (4.94%), *Christensenellaceae_R*-*7_group* (4.65%), *Eubacterium_coprostanoligenes_group* (4.02%), *Bacteroides* (3.85%), *Prevotellaceae_UCG*-*003* (3.55%), *Alistipes* (2.50%), *Ruminococcaceae_UCG*-*013* (2.42%), *Romboutsia* (1.68%), *Paeniclostridium* (1.68%), *Lachnospiraceae_NK3A20_group* (1.64%), *Clostridium_sensu_stricto_1* (1.54%), *Phascolarctobacterium* (1.32%), *Treponema_2* (1.25%), *Family_XIII_AD3011_group* (1.06%), and *Candidatus_Saccharimonas* (1.04%) were the predominant genera (17 in total) and altogether comprised 59.57% of the fecal community (Fig. 2a, b, Table 3).

**Table 3:** Effect of lactation period on diversity at the genus level in the fecal bacterial community

Genera	Stage		SEM	*P* value
	Fresh1d	Fresh14d		
*Ruminococcaceae_UCG*-*005*	12.78	17.02	0.69	0.008
*Rikenellaceae_RC9_gut_group*	6.44	8.61	0.43	0.043
*Christensenellaceae_R*-*7_group*	5.49	3.81	0.27	0.008
*Ruminococcaceae_UCG*-*010*	4.41	5.46	0.32	0.17
*Eubacterium_coprostanoligenes_group*	3.60	4.44	0.23	0.074
*Bacteroides*	3.13	4.58	0.29	0.064
*Romboutsia*	2.51	0.86	0.26	0.008
*Paeniclostridium*	2.33	1.02	0.26	0.043
*Prevotellaceae_UCG*-*003*	2.29	4.81	0.37	0.013
*Alistipes*	1.98	3.03	0.14	0.012
*Lachnospiraceae_NK3A20_group*	1.95	1.34	0.20	0.033
*Ruminococcaceae_UCG*-*013*	1.86	2.98	0.19	0.048
*Family_XIII_AD3011_group*	1.53	0.58	0.14	0.004
*Phascolarctobacterium*	1.35	1.30	0.10	1.000
*Candidatus_Saccharimonas*	1.30	0.78	0.15	0.048
*Treponema_2*	1.19	1.31	0.49	0.008
*Ruminococcus_2*	1.14	0.77	0.12	0.860
*Ruminococcaceae_NK4A214_group*	1.10	0.67	0.10	0.190
*Turicibacter*	1.01	0.33	0.08	0.019
*Ruminococcaceae_UCG*-*014*	0.87	0.95	0.14	0.009
*Lachnospiraceae_AC2044_group*	0.51	0.74	0.08	0.550
*Tyzzerella_4*	0.16	1.08	0.07	0.200

Fresh1d indicates fecal microbiota samples from cows on d1, Fresh14d indicates fecal microbiota samples from cows on d14

### Shared OTUs and core fecal bacteria in fresh cows

Among the total number of OTUs, 116 OTUs were shared by all samples, representing a total relative abundance of 49.82% across samples (Additional file 1: Table S2). At the family level, 18 classified families shared across all samples (Additional file 1: Table S2); of them, the relative abundance > 3% across samples were *Ruminococcaceae* (18.00%), *Lachnospiraceae* (6.52%), *Rikenellaceae* (4.03%), *Bacteroidaceae* (3.46%), *Prevotellaceae* (3.41%) and *Peptostreptococcaceae* (3.36%). In addition, all samples shared two unclassified families belonging to class *Clostridiales* (0.13%) and phyla *Saccharibacteria* (0.78%).

Seven OTUs were classified to the genus *Ruminococcaceae_UCG*-*005*, comprising 13.34% of a total relative abundance, shared among all animals (Additional file 1: Table S2). OTUs belonging to *Bacteroides* (3.46%), *Christensenellaceae_R*-*7_group* (2.62%), *Rikenellaceae_RC9_gut_group* (2.78%) and *unclassified_Lachnospiraceae* (2.66%) each had more than five OTUs shared among all animals. The genera *Acetitomaculum* (0.14%), *Candidatus_Saccharimonas* (0.78%), *Eubacterium_brachy_group* (0.22%), *Mogibacterium* (0.30%), *Prevotellaceae_UCG*-*001* (0.53%), *Prevotellaceae_UCG*-*003* (1.74%), *Ruminococcaceae_UCG*-*010* (0.29%), *Ruminococcaceae_UCG*-*013* (0.86%) and *unclassified_Prevotellaceae* (0.95%) each had two shared OTUs. Finally, there have 17 shared OTUs unclassified at the genus level (6.52%), 8 of which were in *Lachnospiraceae*, 2 in *Prevotellaceae*, 3 in *Ruminococcaceae*, 1 in *Peptococcaceae*,1 in *Porphyromonadaceae*, 1 in *Bacteroidales_RF16_group*, 1 in *Bacteroidales_S24*-*7_group* and 1 in *Clostridiales*.

### Lactation stages induced a variation in fecal bacteria community

At the phylum level, the relative abundance of *Firmicutes*, *Proteobacteria*, *Saccharibacteria* and *Cyanobacteria* was significantly higher (*P* < 0.05) in the Fresh1d than in the Fresh14d group (Table 2). In contrast, *Bacteroidetes* and *Tenericutes* had higher (*P* < 0.01) relative abundance in Fresh14d compared with Fresh1d. Phyla *Actinobacteria* tended to increase (*P* = 0.067) and *Verrucomicrobia* tended to decrease (*P* = 0.067) as lactation progressed.

At the genus level, the relative abundance of *Ruminococcaceae_UCG*-*005*, *Ruminococcaceae_UCG*-*013*, *Prevotellaceae_UCG*-*003*, *Alistipes*, *Ruminococcaceae_UCG*-*013* and *Ruminococcaceae_UCG*-*014* was lower (*P* < 0.05) in the Fresh1d group compared with that in the Fresh14d group (Table 3). In contrast, the abundance of *Christensenellaceae_R*-*7_group*, *Clostridium_sensu_stricto_1*, *Romboutsia*, *Paeniclostridium*, *Lachnospiraceae_NK3A20_group*, *Escherichia*-*Shigella*, *Family_XIII_AD3011_group*, *Candidatus_Saccharimonas*, *Treponema_2* and *Turicibacter* was higher (*P* < 0.01) in the Fresh1d group than in the Fresh14d group.

**Fig. 3:**
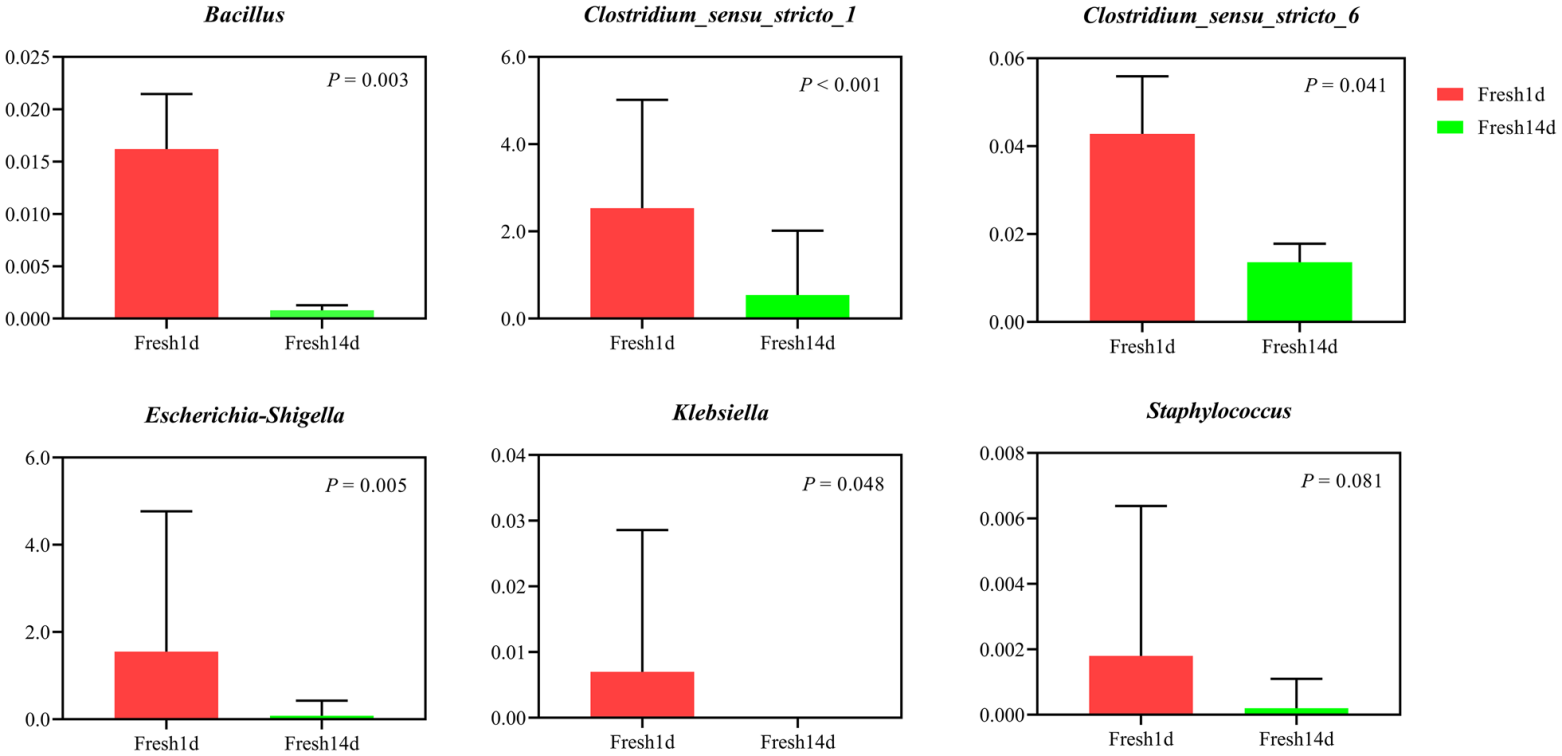
The barplot of the relative abundance of potentially pathogenic bacteria in dairy cows during early lactation. Fresh1d indicates fecal microbiota samples from cows on d1, Fresh14d indicates fecal microbiota samples from cows on d14

The abundance of some potentially pathogenic bacteria was significantly decreased (*P* < 0.05) as lactation progressed, these included *Bacillus*, *Clostridium_sensu_stricto_1*, *Clostridium_sensu_stricto_6* and *Escherichia*-*Shigella* (Fig. 3). More specifically, the relative abundance of *Klebsiella* decreased from 0.007% on Fresh1d to 0% on Fresh14d (*P* = 0.048, Fig. 3). These findings suggest a higher risk of infection in dairy cows after calving and it was decreased as lactation progressed.

## Discussion

The objective of this study was to elucidate the composition and the dynamic changes of the fecal microbiota in dairy cows during early lactation stage using 16S rRNA gene sequencing technology. In this study, we found that the lactation stage of dairy cows significantly contributes to the variation in the fecal bacteria community of lactation cows. Lactation stage was significantly correlated to the relative abundances of the most major and minor contributing phyla. Clustering based on Bray–Curtis distances revealed that samples from Fresh1d clustered away from samples from Fresh14d. Additionally, Fresh1d samples had more OTUs, higher Chao1 and Shannon index in common with Fresh14d samples. Collectively, our data demonstrate that the relative abundance of most major and minor contributing phyla differed by days post-freshening.

Similar to previous pyrosequencing-based studies (Hagey et al. 2019; Huang et al. 2020), the most abundant fecal bacteria at the phylum level that were shared across all samples were the *Firmicutes* and *Bacteroidetes*. We also found that the fecal bacteria community was dynamic over the lactation period, with large discrepancies were observed at the taxonomic level of the phylum and genus, indicating that the adult fecal microbial community with a fluctuation. Recently it was shown that lactation stage has a great impact on shaping the ruminal bacterial community in dairy cows (Bainbridge et al. 2016). Thus, the fecal bacteria difference of samples from Fresh1d and Fresh14d is understandable as they suffer the different lactation stages. To our best knowledge, no other studies have examined the shift in fecal bacterial populations with lactation, although some studies have described the dynamics of rumen bacteria during the transition period. For example, Pitta et al. (2014) described the temporal dynamics of rumen bacteria from five primiparous and five multiparous Holstein dairy cows during the transition period (21 days before parturition, 1–3, 28 and 56 days in milk). In their study, an increase in the abundance of bacteria in the phylum *Bacteroidetes* and a decrease in bacteria from the phylum *Firmicutes* were observed between pre- and post-partum periods in primiparous cows. In according to results from Pitta et al. (2014), a smilar results was observed in our study that an increase in the abundance of bacteria in the phylum *Bacteroidetes* and a decrease in bacteria from the phylum *Firmicutes* were observed from 1 to 14 days after calving. Another explanation is the dietary shift, as cows after calving experienced acute dietary transition and the microbiota adaption needs time. Before calving, cows were fed a low concentrate-to-forage diet and then switch to a high concentrate-to-forage diet. A large body of work points that the composition of the bovine foregut (Clemmons et al. 2019; Henderson et al. 2015) and hindgut microbiota (Kim et al. 2014; Zhang et al. 2018) was shaped by the diet; however, this study was not specifically designed to address the effects of specific feedstuffs on the bovine fecal microbiota. More studies are needed to research the effects of diet on fecal microbiota in lactation dairy cows.

In this study, we define the core taxa in feces as the 48 identified genera present in all samples of fresh cows. Of the two studies that investigated the fecal bacteria of dairy cows, one reported that genera *Clostridium*, *Porphyromonas*, *Bacteroides*, *Ruminococcus*, *Alistipes*, *Lachnospira* and *Prevotella* were shared in all samples of 20 lactating dairy cows (Dowd et al. 2008). The other study found a consistent presence of highly abundant genera-*Prevotella*, *CF231*, *YRC22*, *Parabacteroides*, *5*-*7N15*, *Oscillospira*, *Ruminococcus*, *Clostridium*, *Mogibacterium*, *Coprococcus*, *Butyrivibrio*, *Dorea*, *Turicibacter* and *Treponema*–in 150 lactating dairy cows (Hagey et al. 2019). While some families of all these genera are present in our core fecal bacteria, *Paraprevotellaceae*, *Spirochaetaceae*, *Coriobacteriaceae*, *Turicibacteraceae* and *Mogibacteriaceae* are not the part of our definition of core. Also, of the 13 genera shared in the 20 lactating dairy cows in their study, *Bacteroides*, *Ruminococcus*, *Alistipes*, *Lachnospiraceae*, *Prevotella* and *Anaerotruncus* were the core genera that we observed in 20 fresh cows. Together, these observations suggest that there may be a highly conserved core microbiota defined by the highly abundant genera for the fresh dairy cow feces. However, further studies are required to confirm and understand the function of the fecal microbiome in lactating dairy cows using metagenomic and transcriptomic methods.

Gram-positive rod-shaped *Bacillus* species can persist for many years in soil (Manyi-Loh et al. 2016) and cause clinical mastitis in dairy cows (Yu et al. 2006). The class *Clostridia* consists of gram-positive anaerobic spore-forming bacteria that are ubiquitous in the gastrointestinal tract (Girija et al. 2013). Some species of *Clostridia* have been linked with mastitis, blackleg, hemoglobinuria, malignant edema and infant botulism both in cattle and humans (Williams 2014). *Streptococcus* are gram-positive, facultatively anaerobic, lactic-acid producing commensal bacteria present in the gastrointestinal tract of humans and animals (Silva et al. 2011). Members of this genus can produce virulence factors and express antibiotic-resistance genes in fresh and dry cattle manure (Eaton and Gasson 2001; Franz et al. 2001). In recent years, *Klebsiella* spp. mastitis has become a serious problem in herds because of using sand and dry feces bedding, this is attributed to fecal shedding of *Klebsiella* spp. by healthy adult dairy cows (Munoz et al. 2006). *Klebsiella* spp. is one of the main gram-negative pathogens (Olde Riekerink et al. 2008) that causes mastitis, a decrease in milk production, culling of dairy cows (Gröhn et al. 2004; Wilson et al. 2007) and antimicrobial resistance (Roberson et al. 2004). Similar to functional prediction, precise detection of pathogens was unfeasible with our data. Despite the fact that the relative abundance of genera commonly associated with pathogenic bacteria (genus *Bacillus*, *Clostridium_sensu_stricto_1*, *Clostridium_sensu_stricto_6* and *Escherichia*-*Shigella*) decreased from d1 to d14 after freshening, we cannot say conclusively if these are pathogenic or commensal. Thus, PCR and culture-based methods remain the gold standard for routine detection of these pathogens.

In conclusion, we observed significant changes in alpha diversity and relative abundance of fecal microbial taxa at the phylum and genus level using 16S rRNA gene amplicon sequencing analyses, in response to the dietary and lactation period perturbations encountered by dairy cows during early lactation period. Moreover, we found a decrease in genera associated with pathogenic bacteria as lactation progressed during early lactation onward. In accordance with a clear shift in the fecal bacteria after NMDS analyses, we observed significant changes in the relative abundance of the dominant phyla and genera in fecal bacteria as lactation progressed. Further research should focus on the impact of pathogenic and environmental factors on fecal microbial communities and the health of dairy cows.

## Supplementary information

**Additional file 1: Table S1.** Ingredients and chemical composition of the experimental diets (as dry matter basis). **Table S2.** Shared bacterial OTUs and taxa among all samples.

## Data Availability

All the raw DNA sequences were deposited in the National Center for Biotechnology Information Sequence Read Archive database and are publicly accessible under the accession number PRJNA628713. The data set supporting the conclusions of this article is included within the article.
