# Correction to: Dynamic changes of the fecal bacterial community in dairy cows during early lactation

**DOI:** 10.1186/s13568-021-01185-w

**Published:** 2021-03-06

**Authors:** Shuai Huang, Shoukun Ji, Feiran Wang, Jie Huang, Gibson Maswayi Alugongo, Shengli Li

**Affiliations:** 1 The State Key Laboratory of Animal Nutrition, Beijing Engineering Technology Research Center of Raw Milk Quality and Safety Control, College of Animal Science and Technology, China Agricultural University, Beijing, 100193 China; 2 College of Animal Science and Technology, Hebei Agricultural University, Baoding, 071001 China; 3 College of Animal Science and Technology, Yangzhou University, Yangzhou, 225009 China

## Correction to: AMB Expr (2020) 10:167 10.1186/s13568-020-01106-3

Following publication of the original article (Huang et al. 2020), the authors identified an error in the figure parts. Figure 3 contains only two groups-Fresh1d and Fresh14d. But the published version has three groups (Fresh1d, Fresh14d and mid) and incorrect p values. The corrected Fig. 3 is presented with this erratum.

**Fig. 3:**
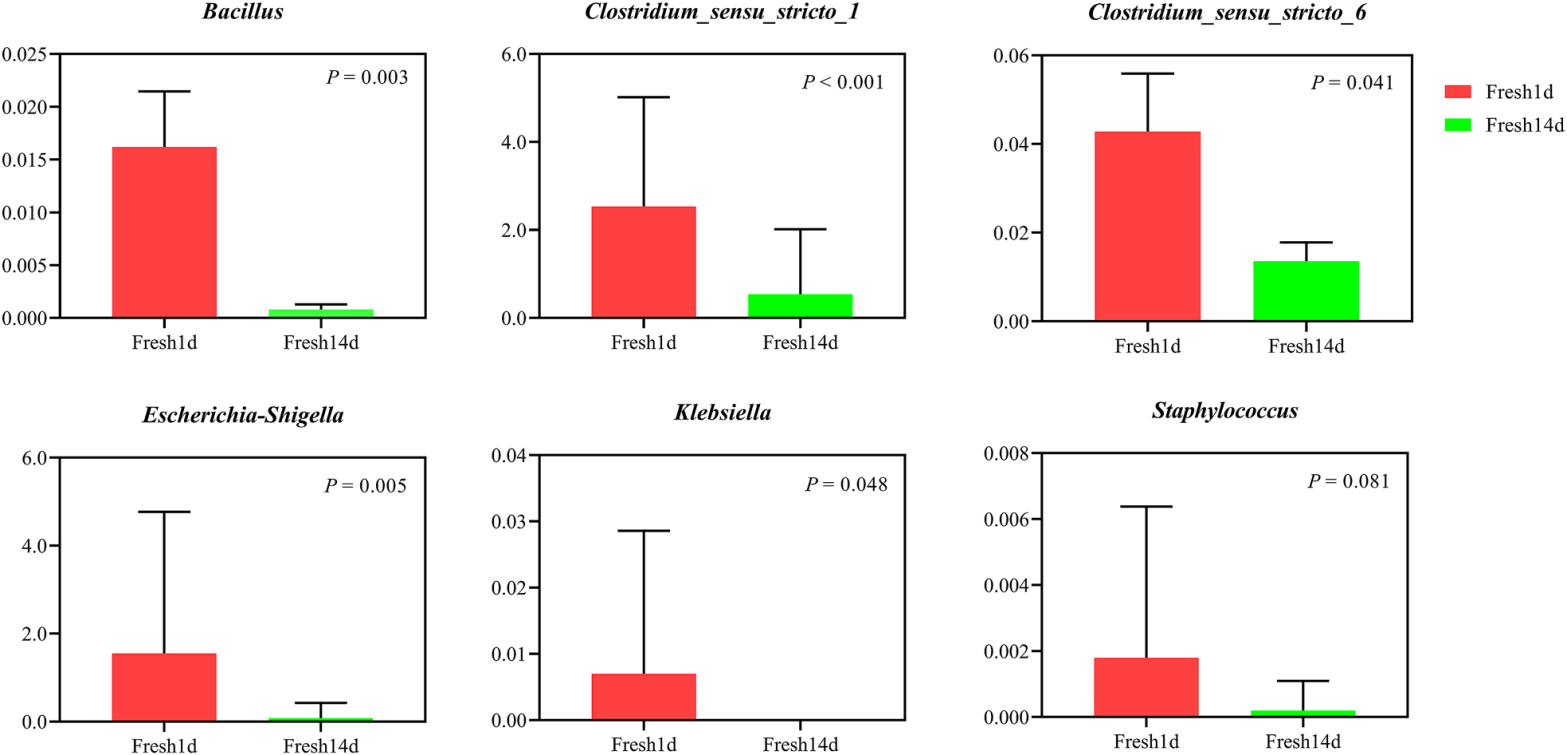
The barplot of the relative abundance of potentially pathogenic bacteria in dairy cows during early lactation. Fresh1d indicates fecal microbiota samples from cows on d1, Fresh14d indicates fecal microbiota samples from cows on d14

The original article (Huang et al. 2020) has been updated.
