# Supplementary material for: Dynamic changes of the fecal bacterial community in dairy cows during early lactation
Source: AMB Express. 2020 Sep 17;10:167. doi: 10.1186/s13568-020-01106-3 (PMC7498527; doi:10.1186/s13568-020-01106-3)
Supplement: Supplementary file 1 — Additional file 1: Table S1. Ingredients and chemical composition of the experimental diets (as dry matter basis). Table S2. Shared bacterial OTUs and taxa among all samples. [file 13568_2020_1106_MOESM1_ESM.docx]

**Table S1.** Ingredients and chemical composition of the experimental diets (as dry matter basis).

| Items^1^ | Fresh |
| --- | --- |
| **Ingredients, kg** |  |
| Oat grass | 0.59 |
| Alfalfa hay | 1.48 |
| Corn silage | 5.52 |
| Corn | 0.58 |
| Soybean meal | 1.46 |
| Soybean hull | 0.99 |
| Sprayed corn husk | 0.09 |
| DDGS | 0.74 |
| 5% premix | 0.32 |
| Flaked corn | 0.99 |
| Cottonseed | 0.25 |
| 5% anion premix | --- |
| Molasses | 0.25 |
| Additives | 0.10 |
| Sodium bicarbonate | --- |
| **Contents, %** |  |
| Dry matter as fed | 49.32 |
| Crude protein | 16.56 |
| Fat | 3.4 |
| ADF | 20.58 |
| NDF | 34.63 |

^1^DDGS dried distillers grains with solubles, DM dry matter, NDF neutral detergent fiber, ADF acid detergent fiber, NFC nonfiber carbohydrates.

**Table S2.** Shared bacterial OTUs and taxa among all samples.

| Phylum | Family | Genus (OTU) | Relative abundance (%) |
| --- | --- | --- | --- |
| ***Bacteroidetes*** | *Bacteroidaceae* | *Bacteroides* (OTU286, OTU308, OTU315, OTU319, OTU325, OTU330, OTU366, OTU423) | 3.46 |
|  | *Bacteroidales_Incertae_Sedis* | *Phocaeicola* (OTU358) | 0.12 |
|  | *Bacteroidales_RF16_group* | *unclassified_Bacteroidales_RF16_group* (OTU445) | 1.52 |
|  | *Bacteroidales_S24-7_group* | *unclassified*_*Bacteroidales_S24-7_group* (OTU566) | 0.32 |
|  | *Porphyromonadaceae* | *Parabacteroides* (OTU281) | 0.21 |
|  |  | *unclassified_Porphyromonadaceae* (OTU347) | 0.21 |
|  | *Prevotellaceae* | *Alloprevotella* (OTU300) | 0.18 |
|  |  | *Prevotellaceae_UCG-001* (OTU409, OTU421) | 0.53 |
|  |  | *Prevotellaceae_UCG-003* (OTU293, OTU472) | 1.74 |
|  |  | *unclassified*_*Prevotellaceae* (OTU313, OTU310) | 0.95 |
|  | *Rikenellaceae* | *Alistipes* (OTU278, OTU314, OTU318, OTU469) | 1.25 |
|  |  | *Rikenellaceae_RC9_gut_group* (OTU419, OTU420, OTU448, OTU499, OTU1635, OTU504, OTU513, OTU523) | 2.78 |
| ***Firmicutes*** | *Acidaminococcaceae* | *Phascolarctobacterium* (OTU16) | 1.32 |
|  | *Christensenellaceae* | *Christensenellaceae_R-7_group* (OTU1016, OTU1062, OTU1189, OTU1345, OTU1635, OTU642, OTU665, OTU669, OTU710, OTU716) | 2.62 |
|  | *Clostridiaceae_1* | *Clostridium_sensu_stricto_1* (OTU1440) | 1.22 |
|  | *Defluviitaleaceae* | *Defluviitaleaceae_UCG-011* (OTU1366) | 0.05 |
|  | *Erysipelotrichaceae* | *Turicibacter* (OTU30) | 0.67 |
|  | *Family_XIII* | *Anaerovorax* (OTU1128) | 0.08 |
|  |  | *Eubacterium_brachy_group* (OTU1127, OTU1508) | 0.22 |
|  |  | *Eubacterium_nodatum_group* (OTU1283) | 0.32 |
|  |  | *Family_XIII_AD3011_group* (OTU1372, OTU1743, OTU1979, OTU2073) | 0.61 |
|  |  | *Mogibacterium* (OTU1153, OTU1159) | 0.30 |
|  | *Lachnospiraceae* | *Acetitomaculum* (OTU1573, OTU1612) | 0.14 |
|  |  | *Blautia* (OTU1577) | 0.11 |
|  |  | *Coprococcus_3* (OTU1724) | 0.06 |
|  |  | *Dorea* (OTU1504) | 0.25 |
|  |  | *Eubacterium_hallii_group* (OTU1707) | 0.04 |
|  |  | *Lachnoclostridium_10* (OTU602) | 0.16 |
|  |  | *Lachnospiraceae_AC2044_group* (OTU1637) | 0.40 |
|  |  | *Lachnospiraceae_NK3A20_group* (OTU583, OTU585, OTU592, OTU595) | 1.60 |
|  |  | *Lachnospiraceae_UCG-010* (OTU649) | 0.23 |
|  |  | *Marvinbryantia* (OTU1596) | 0.08 |
|  |  | *Ruminococcus_gauvreauii_group* (OTU1572) | 0.17 |
|  |  | *Tyzzerella_4* (OTU1434) | 0.62 |
|  |  | *unclassified_Lachnospiraceae* (OTU1012, OTU1263, OTU1462, OTU1478, OTU1553, OTU1600, OTU1614, OTU1632) | 2.66 |
|  | *Peptostreptococcaceae* | *Paeniclostridium* (OTU1797) | 1.68 |
|  |  | *Romboutsia* (OTU1802) | 1.68 |
|  | *Peptococcaceae* | *unclassified_Peptococcaceae* (OTU2) | 0.22 |
|  | *Ruminococcaceae* | *Anaerotruncus* (OTU1354) | 0.11 |
|  |  | *Candidatus_Soleaferrea* (OTU1944) | 0.23 |
|  |  | *Eubacterium_coprostanoligenes_group* (OTU659, OTU673, OTU685, OTU763) | 0.89 |
|  |  | *Flavonifractor* (OTU687) | 0.35 |
|  |  | *Ruminococcaceae_NK4A214_group* (OTU681, OTU695, OTU702) | 0.46 |
|  |  | *Ruminococcaceae_UCG-002* (OTU987) | 0.26 |
|  |  | *Ruminococcaceae_UCG-004* (OTU662) | 0.45 |
|  |  | *Ruminococcaceae_UCG-005* (OTU1323, OTU1483, OTU618, OTU683, OTU694, OTU724, OTU792) | 13.34 |
|  |  | *Ruminococcaceae_UCG-010* (OTU674, OTU703) | 0.29 |
|  |  | *Ruminococcaceae_UCG-013* (OTU1357, OTU1379, OTU1497, OTU658) | 0.86 |
|  |  | *Ruminococcaceae_UCG-014* (OTU1269) | 0.14 |
|  |  | *Ruminococcus_2* (OTU1588) | 0.10 |
|  |  | *unclassified_* *Ruminococcaceae* (OTU953, OTU1951, OTU1948) | 0.51 |
|  | *unclassified* _*Clostridiales* | *unclassified_Clostridiales* (OTU1010) | 0.13 |
| ***Proteobacteria*** | *Alcaligenaceae* | *Sutterella* (OTU22) | 0.09 |
| ***Saccharibacteria*** | *unclassified_* *Saccharibacteria* | *Candidatus_Saccharimonas* (OTU1166, OTU1178) | 0.79 |
